# Supplementary material for: Plasmid-mediated metronidazole resistance in Clostridioides difficile
Source: Nat Commun. 2020 Jan 30;11:598. doi: 10.1038/s41467-020-14382-1 (PMC6992631; doi:10.1038/s41467-020-14382-1)
Supplement: Supplementary file 1 — Supplementary information [file 41467_2020_14382_MOESM1_ESM.pdf]

SUPPLEMENTARY MATERIAL

Plasmid-mediated metronidazole resistance in *Clostridioides difficile*

Boekhoud *et al.*

Supplementary Methods

Conjugation of pCD-METRO between *C. difficile* strains

A spontaneous rifampicin resistant 630Δ*erm* strain was generated by incubating cells on a BHI agar plate supplemented with 25 µg/ml rifampicin until colonies appeared. The resulting strain EVE17 was found to have acquired the well described R505K mutation in *rpoB* as verified by Sanger sequencing using primers oIB-103, oIB-104, oIB-105, oIB-106, oIB-107 and oIB-108[1]. Conjugative transfer was attempted between a pCD-METRO harboring RT010 strain IB138 and CD37[2], IB140, EVE17 or WKS1710 (630Δ*erm tcdA*::CT *tcdB*::CT; Li^R)[3]. IB138 was chosen as a donor as this strain only carries the pCD-METRO plasmid without any other extrachromosomal elements. CD37 is a strain frequently used in filter-mating experiments[2,4]. IB140 is a pCD-METRO negative strain that we expect to be conducive to acquisition of the plasmid (Table 1, Supplementary Table 1). To exclude the possibility of rifampicin selection interfering with the conjugation process, WKS1710 (630Δ*erm tcdA*::CT *tcdB*::CT) was also used as a recipient. Transconjugants were selected using 4 µg/ml metronidazole combined with either 20 µg/ml rifampicin (for CD37 and EVE17) or 20 µg/ml lincomycin (for WKS1710). Though we cannot exclude the possibility that intraspecies transfer is possible between strains that were not tested, or under conditions different from the ones we employed here, we have not been able to observe transfer of pCD-METRO from RT010 into our lab strains under the conditions used.

Serial passaging of pCD-METRO containing strains

To try to cure plasmid-containing strains, IB125 (RT012 $pCD\text{-}METRO^{shuttle}$; MTZ^R) and IB134 (RT020 pCD-METRO; MTZ^R) were cultured on BHIY agar plates supplemented with CDSS. From this plate, a liquid culture (BHIY/CDSS) was inoculated. After 24h of growth (an estimated 12 doublings), the culture was 1/200 diluted into fresh medium. The same procedure was followed 2 more times. After this (an estimated number of doublings >50), the culture was serially diluted and plated onto

BHIY/CDSS agar plates. Individual colonies (n=200) were screened for resistance against thiamphenicol (indicative of the presence of the pCD-METRO^{shuttle} vector) and metronidazole (to assess the metronidazole resistance phenotype) by patching colonies onto selective agar media. We found that all colonies screened were resistant against both antimicrobials, suggesting that pCD-METRO^{shuttle} was not lost. We also attempted curing by repeated sub-culturing (5x) on solid BHI/CDSS agar plates. We did not attempt to increase the chance of plasmid-loss using mutagens, as the results obtained from such experiments would be difficult to interpret. Thus, pCD-METRO is not readily lost by culturing on media without metronidazole.

Supplementary Figures

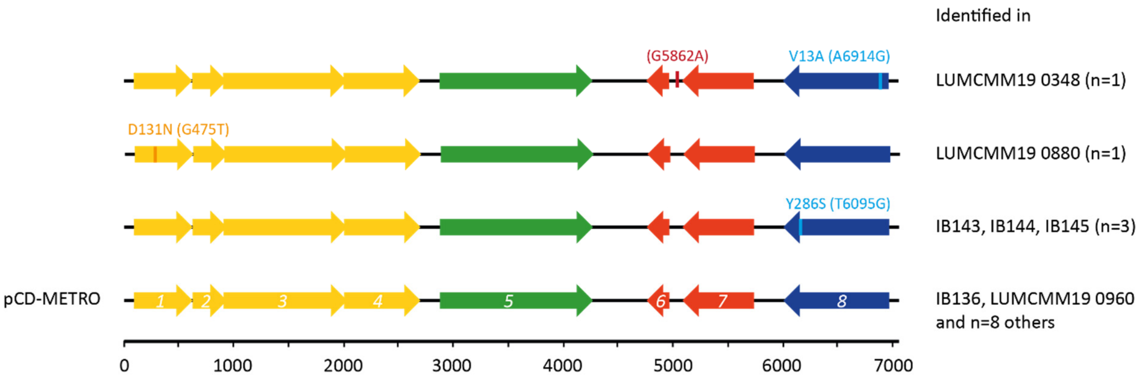

Supplementary Figure 1. Variants of pCD-METRO as identified in this study. Assembly and SNP calling was performed as described in Supplementary Methods. Strain IB136 is the reference for pCD-METRO, and most strains are similar to this plasmid. Variants are indicated with vertical bars. Amino acid changes are indicated in single letter genetic code for non-synonymous substitutions (in ORFs). Nucleotide changes are given in between brackets with the relevant nucleotide position in relation to the scale bar below (forward strand).

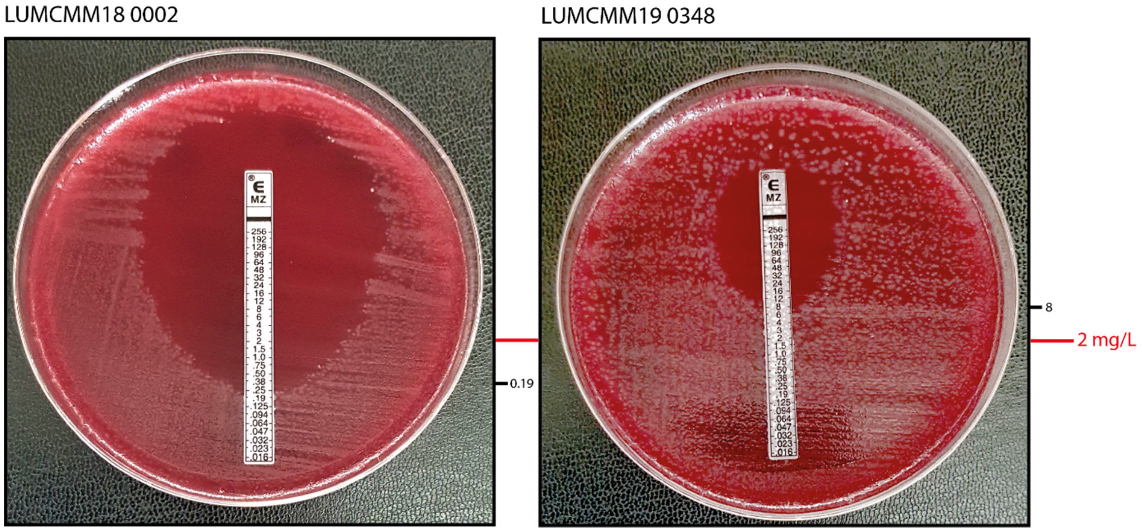

Supplementary Figure 2. Metronidazole resistance of RT020 strains obtained from routine surveillance. Strains LUMC19 0348 (pCD-METRO negative) and LUMC19 0830 (pCD-METRO positive) from the patient were tested for metronidazole resistance using E-test. 2 mg/L is the EUCAST epidemiological cut-off for metronidazole that was used to define resistance in this study[5]. Source data are provided as a Source Data file.

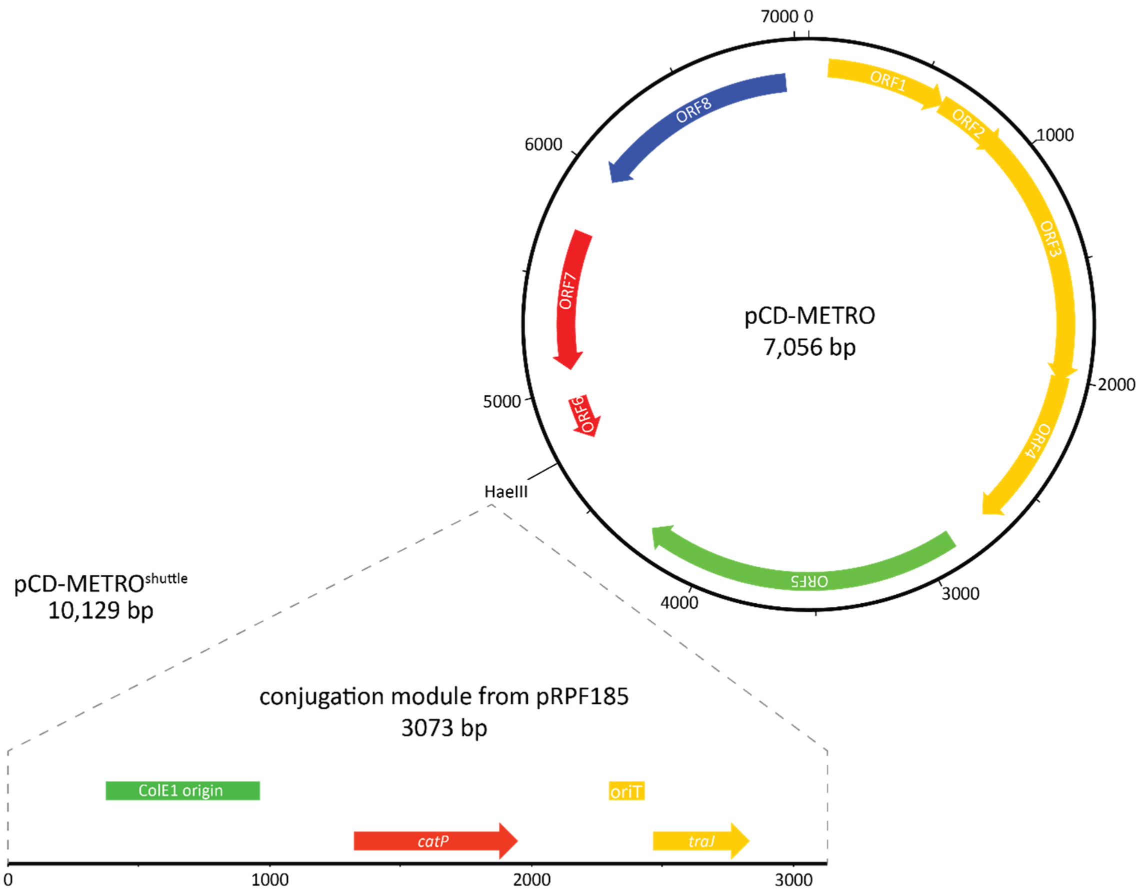

Supplementary Figure 3. Schematic representation of the construction of pCD-METRO^{shuttle} (pIB86): the PCR-amplified conjugation module from pRPF185 was inserted in the unique HaeIII restriction site through Gibson assembly as described in Supplementary Methods.

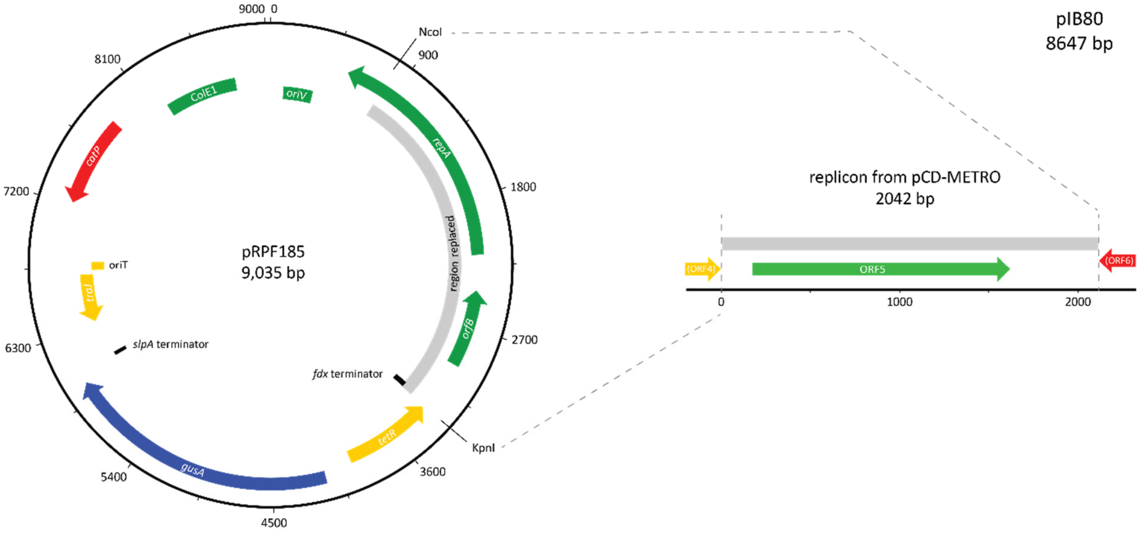

Supplementary Figure 4: pIB80 was generated by cloning the region containing the ORF encoding the putative replication protein from pCD-METRO into the indicated area of pRPF185, disrupting the pCD6 replicon (*orfB* + *repA*), as indicated in the Supplementary Methods.

Supplementary Tables

Name	Relevant features	Source/reference
pRPF185	tetR P_{tet}-s*luc*^{opt}; catP	A Oliveira Paiva et al., 2016
pCD-METRO	(see: accession number ERZ807316)	This study
pIB20	pRPF185 P_{cd0716}-s*luc*^{opt}; catP	This study
pIB76	tetR Ptet-nimB; catP	This study
pIB80	tetR P_{tet}-*gusA -pCD-METROreplicon*	This study
pIB86	pCD-METRO containing shuttle fragment; pCD-METRO-*oriT-pBR322-traJ catP*; catP	This study

Supplementary table 1: plasmids used in this study

**Name**	**Sequence (5’->3’)**	**Description**	**Source**
oBH-1	CCTCGTAGAATCCGGTGCAA	Forward primer annealing to *orf6* of pCD-METRO	This study
oBH-2	TATTTCCTTGCCGCTGAGGT	Reverse primer annealing to *orf6* of pCD-METRO	This study
oBH-3	GCAGAGCGTTGTGGTATTCG	Forward primer annealing to *orf6* of pCD-METRO	This study
oBH-4	GTATTTCCTTGCCGCTGAGG	Reverse primer annealing to *orf6* of PCD-METRO	This study
oBH-5	AGTAGGCGGTCGGTACTTCT	Forward primer annealing to *orf5* of pCD-METRO	This study
oBH-6	CTTCCGTTCCGCTTGTTTGG	Reverse primer annealing to *orf5* of pCD-METRO	This study
oBH-7	GGCTCAGACACTTCTACCGC	Forward primer annealing to *orf7* of pCD-METRO	This study
oBH-8	CCCCTTCCAGGGTGTTTTCT	Reverse primer annealing to *orf7* of pCD-METRO	This study
oBH-9	GGGCTATAAGACCGACTGGC	Forward primer annealing to *orf3* of pCD-METRO	This study
oBH-10	AACGGTCTCTACCTCCGTCA	Reverse primer annealing to *orf3* of pCD-METRO	This study
oBH-11	ACTTACACTGCAAACGGTGC	Forward primer annealing to *orf8* of pCD-METRO	This study
oBH-12	TCGTTGCTTGTGAGGTGAGT	Reverse primer annealing to *orf8* of pCD-METRO	This study
oIB-103	CATATAAGATAAAAAATATATTGAAAAATAC	Forward primer used for sequencing *rpoB*	This study
oIB-104	GATATGACCTAGCAAAAGTTGGTAG	Forward primer used for sequencing *rpoB*	This study
oIB-105	AGAATCACCATATAGAAAATTTGATAAAG	Forward primer used for sequencing *rpoB*	This study
oIB-106	GGAGAAACTGAACTTACTGCTGAG	Forward primer used for sequencing *rpoB*	This study
oIB-107	GACAGATGAAGACCAAGAAATAGAAG	Forward primer used for sequencing *rpoB*	This study
oIB-108	ATTGTCTTATTTTTTCTGGAGAAGCC	Forward primer used for sequencing *rpoB*	This study
oIB-120	GCCGAAAAAGAAAAACTGCCGGG	Sequencing primer for pCD-METRO^{shuttle}	This study
oIB-121	CTTACTCAAACGGAAGTGATGAGAG	Sequencing primer for pCD-METRO^{shuttle}	This study
oIB-122	GCGTTTATAGTACACTGGCTTGTG	Sequencing primer for pCD-METRO^{shuttle}	This study
RP314	GAAGGTTGACCACGGTATCAT	Forward qPCR primer for *catP*	EM Ransom et al., 2015
RP315	CGCAACGGTATGGAAACAATC	Reverse qPCR primer for *catP*	EM Ransom et al., 2015
TEQ009	AAGAGCTGGATTCGAAGTGCGTGA	Forward qpCR primer for *rpoB*	EM Ransom et al., 2015
TEQ010	ACCGATATTTGGTCCCTCTGGAGT	Reverse qPCR primer for *rpoB*	EM Ransom et al., 2015
oWKS-1070	GTCTTGGATGGTTGATGAGTAC	Forward primer in *gluD*	WK Smits et al., 2018
oWKS-1071	TTCCTAATTTAGCAGCAGCTTC	Reverse primer in *gluD*	WK Smits et al., 2018
oWKS-1241	CACCGACGAGCAAGGCAAGACCG	Sequencing primer for pCD-METRO^{shuttle}	E Van Eijk et al., 2019
oWKS-1387	CAGATGAGGGCAAGCGGATG	Sequencing primer for pCD-METRO^{shuttle}	This study
oWKS-1388	CGTCGGTGAGCCAGAGTTTC	Sequencing primer for pCD-METRO^{shuttle}	This study
oWKS-1537	TAGGGTAACAAAAAACACCG	Sequencing primer for pCD-METRO^{shuttle}	PT van Leeuwen et al., 2016
oWKS-1539	GGATTTCACATTTGCCGTTTTGTAAAC	Sequencing primer for pCD-METRO^{shuttle}	PT van Leeuwen et al., 2016
oWKS-1540	GATCTTTTCTACGGGGTCTGAC	Sequencing primer for pCD-METRO^{shuttle}	PT van Leeuwen et al., 2016
oWKS-1574	AAACAAACCACCGCTGGTAG	Sequencing primer for pCD-METRO^{shuttle}	This study
oWKS-1656	TAGCGGATCCAAGCGTTCTGAACGCACTG	Sequencing primer for pCD-METRO^{shuttle}	This study
oWKS-1658	TAGCGGATCCGGGCTTACTTCTGGGTATCC	Sequencing primer for pCD-METRO^{shuttle}	This study
oWKS-1659	TAGCGGTACCTGTTGCCTGCTTCCTGTATG	Sequencing primer for pCD-METRO^{shuttle}	This study
oWKS-1661	TAGCGGTACCATCCACGCACCACAACAC	Sequencing primer for pCD-METRO^{shuttle}	This study
oWKS-1663	CAAGACGGTCGGCGTTGCGCTCGAAGATGGATAAAATAAAATAGAGGCTATAAATAGC	Forward primer for PCR of shuttle fragment *pBR322ori-catP-oriT-traJ*	This study
oWKS-1664	CGATAAATCTTGATTTGATGAAGTACAAGGTTAGTAGGTGCTTTTTTAAAAC	Reverse primer for PCR of shuttle fragment *pBR322ori-catP-oriT-traJ*	This study
oWKS-1678	CCATCTTCGAGCGCAACG	Sequencing primer for pCD-METRO^{shuttle}	This study

Supplementary table 2: oligonucleotides used in this study

ANOVA table	SS	DF	MS	F (DFn, DFd)	P value
Row Factor	6,189	2	3,094	F (2, 6) = 1,344	P=0,3294
Column Factor	2945	3	981,8	F (3, 6) = 426,4	P<0,0001
Residual	13,82	6	2,303		

Tukey's multiple comparisons test	Mean Diff,	95,00% CI of diff,	Significant?	Summary	Adjusted P Value
630Δ*erm* vs. IB30	-3,543	-7,832 to 0,7457	No	ns	0,1018
630Δ*erm* vs. IB90	-25,25	-29,54 to -20,96	Yes	****	<0,0001
630Δ*erm* vs. IB125	-38,07	-42,36 to -33,78	Yes	****	<0,0001
IB30 vs. IB90	-21,71	-26,00 to -17,42	Yes	****	<0,0001
IB30 vs. IB125	-34,53	-38,82 to -30,24	Yes	****	<0,0001
IB90 vs. IB125	-12,82	-17,11 to -8,531	Yes	***	0,0002

Supplementary Table 3. Statistics associated with the qPCR analysis. These data relate to Figure 7A of the main text of the manuscript.

Supplementary Files

Supplementary File 1. GenBank formatted sequence of pIB86 (pCD-METRO^{shuttle})

```
LOCUS       pIB86                 10129 bp    DNA     circular SYN 19-DEC-2019
DEFINITION  pCD-METRO with shuttle module inserted in HaeIII site.
ACCESSION   pCD-METRO-
KEYWORDS    .
SOURCE      Unknown
  ORGANISM  Unknown
            Unclassified.
REFERENCE   1  (bases 1 to 10129)
  AUTHORS   Boekhoud et al (2019)
  JOURNAL   Nature Communications
FEATURES             Location/Qualifiers
     primer_bind     1..22
                     /standard_name="oWKS-1677"
     CDS             complement(25..225)
                     /gene="IB136_3862"
                     /product="FMN-binding split barrel/nimB-like, partial"
                     /standard_name="ORF6"
     primer_bind     36..55
                     /standard_name="oBH-4"
     primer_bind     37..56
                     /standard_name="oBH-2"
     misc_feature    complement(174..193)
                     /note="Geneious type: primer_bind_reverse"
                     /standard_name="oBH-1"
     misc_feature    complement(204..223)
                     /note="Geneious type: primer_bind_reverse"
                     /standard_name="oBH-3"
     misc_feature    complement(239..252)
                     /standard_name="Putative RBS"
     CDS             complement(355..1002)
                     /gene="IB136_3863"
                     /product="Metallo-hydrolase/oxidoreductase superfamily"
                     /standard_name="ORF7"
     primer_bind     492..511
                     /standard_name="oBH-8"
     misc_feature    complement(858..877)
                     /note="Geneious type: primer_bind_reverse"
                     /standard_name="oBH-7"
     CDS             complement(1268..2227)
                     /gene="IB136_3864"
                     /product="Transposase, Tn5-like, core"
                     /standard_name="ORF8"
     primer_bind     1795..1814
                     /standard_name="oBH-12"
     misc_feature    complement(1968..1987)
                     /note="Geneious type: primer_bind_reverse"
                     /standard_name="oBH-11"
     CDS             2417..2947
                     /gene="IB136_3857"
                     /product="mutS2 family; MutS_III SF; possible DNA mismatch
                     binding protein"
                     /standard_name="ORF1"
     primer_bind     2586..2610
                     /standard_name="oIB-121"
     CDS             2940..3260
                     /gene="IB136_3858"
                     /product="MobC-like relaxase/Arc-type ribbon-helix-helix"
                     /standard_name="ORF2"
     CDS             3221..4348
                     /gene="IB136_3859"
```

```
                     /product="Endonuclease relaxase, MobA/VirD2"
                     /standard_name="ORF3"
     primer_bind     3865..3884
                     /standard_name="oBH-9"
     CDS             4326..5027
                     /gene="IB136_3860"
                     /product="Putative replication protein"
                     /standard_name="ORF4"
     misc_feature    complement(4327..4346)
                     /note="Geneious type: primer_bind_reverse"
                     /standard_name="oBH-10"
     misc_feature    4731..4761
                     /standard_name="Design primer here"
     primer_bind     4731..4754
                     /standard_name="oIB-122"
     CDS             5200..6597
                     /gene="IB136_3861"
                     /product="Putative replication protein"
                     /standard_name="ORF5"
     primer_bind     6111..6130
                     /standard_name="oBH-5"
     misc_feature    complement(6364..6383)
                     /note="Geneious type: primer_bind_reverse"
                     /standard_name="oBH-6"
     primer_bind     7027..7084
                     /standard_name="oWKS-1663"
     misc_feature    complement(7039..7056)
                     /note="Geneious type: primer_bind_reverse"
                     /standard_name="oWKS-1678"
     misc_feature    7057..10029
                     /standard_name="shuttle module from pRPF185"
     misc_feature    complement(7198..7220)
                     /note="Geneious type: primer_bind_reverse"
                     /standard_name="oWKS-1241"
     CDS             complement(7323..7694)
                     /product="conjugation protein"
                     /standard_name="traJ"
     primer_bind     7445..7464
                     /standard_name="oWKS-1388"
     misc_feature    complement(7692..7711)
                     /note="Geneious type: primer_bind_reverse"
                     /standard_name="oWKS-1387"
     oriT            7727..7796
     primer_bind     8136..8155
                     /standard_name="oWKS-1537"
     CDS             complement(8212..8835)
                     /gene="catP"
                     /product="chloramphenicol resistance protein"
                     /standard_name="CatP"
     misc_feature    complement(8239..8265)
                     /note="Geneious type: primer_bind_reverse"
                     /standard_name="oWKS-1539"
     primer_bind     8960..8982
                     /standard_name="oIB-120"
     misc_feature    complement(9183..9802)
                     /note="Geneious type: Origin of Replication"
                     /standard_name="pBR322 origin"
     primer_bind     9798..9819
                     /standard_name="oWKS-1540"
     misc_feature    complement(9987..10004)
                     /note="Geneious type: primer_bind_reverse"
                     /standard_name="oWKS-1366"
     misc_feature    complement(10108..30)
                     /note="Geneious type: primer_bind_reverse"
                     /standard_name="oWKS-1664"
ORIGIN
        1 ccttgtactt catcaaatca agatttatcg ttcctgtatt tccttgccgc tgaggtactc
       61 cacttctatt tcaaaaattt gcacagcctt tcctgcgtgg gcaatttcct catcaactaa
```

121 ctgttcgctg ggatagtatt tcattgcaaa tcgttttaac agagttgttg ctcttgcacc
181 ggattctacg aggtgacatc tgccgaatac cacaacgctc tgcacaaacg gtgcccaatc
241 ttcttctttt ccgtaatcgg cacctttcct ctatgaagga tgatttttag ttgaaaaaag
301 cagtctttta tttaaagact gcttcctatt aattctcttc aattttaaat tattttagtt
361 ctttgacatt attgctatac attcaccatg tcccgttgct ataaacttta tatcttcttt
421 ctttaaatct cctataagct tttctctttt ttctgtatca gggatttgtt cttttgttat
481 tctatccagt tccccttcca gggtgttttc taatatttct ccacctgaat taccaaacct
541 aatcattaga tcacttgaaa ataatatttt cctttccaaa tctaaaagta atagcccttc
601 ccataaatgc atttctgaag gatagcttat aaatttgaat tctgtttctc ctattttcat
661 tttttcttca ggtcttttta tcagtacatc tccagctatt ccaaatccat ctaattgtct
721 tgcagtaact tctgaagcaa ttaccctggc atcaggaaat tcttttaata ttatttctag
781 tcccccacac tcatccgact caaaatggga aacaaaaata tacttcagtt tcctgcccat
841 aagtacatcc ttaatttgcg gtagaagtgt ctgagcctgt tgcctgcttc ctgtatgaat
901 taatatgggt tcatctatat ttaataaata ctgatgaaag gttaaatcta ttggtggtgc
961 atagctggtg aattgatata aatcatcgaa tatttgtggc atttaattta cctcctattg
1021 tttactaaaa ttattttcaa actactccaa ttatatactc cctaaattcc acgtgtgttt
1081 tttattagct tcaaaaatca ctatttcacg aagaatttag actgcttctc acacattgta
1141 acattattta caaccacctt tcaatcattt ttgataaatc attgatttca tctttgctgc
1201 aatgatactt aataaactct gcaagttatc cacagagcaa cactcaattt tattgatgat
1261 attcttatta taccagacat ttttcataca ctcccttgta cggatagttt tccgacaact
1321 tcatgattac atatcttgcg gttttgatta tttttgctgc aagaaataca tacttcaaac
1381 gaaaggtctt tatttgctgt ctgtattctg aaggctctaa ggaatcaatc ttgaacaaca
1441 aaaacaggtt atatgaaagc atcatcattt gaaacaccgc ttcattcgcc caaaatgatt
1501 taagtaaaag atgacccacc gccatgtcgt atttggcttc tttaatatag ttctcagcat
1561 tgccacgttt ttcataggat ataactactt tttcagaaag caaggtagta tttgttacaa
1621 agaaaaagta gtcgtattcg gaaccttcta aaagtgataa ttgtgctctt tctttttctg
1681 gtttcagtac gcgagatacg acaaatcttc tgtctttttc ccatttaact aattttgtat
1741 acagttctgt agtttctcta ccttcttctc ctttaacgaa tacaattgat gaattcgttg
1801 cttgtgaggt gagtgtagaa taacttttgg ctttaattaa atatttgcat ccaagagatt
1861 ctatcgtttc gataattttt tcatcaaagt agccactatc cattcgaaat aaaatttcta
1921 aatcgtctga tttgatgtta gcaacaattt ctttgatcat ttccgcagca ccgtttgcag
1981 tgtaagtatt gccacttctt acaaatccgg taacatatgc ttttaattcg tcgcaaaatg
2041 caaattggat attgtagcat cggtttccca gtttcttagg attatatcct tttgacgcac
2101 cttcttgatg accttctacg ttaattacac tactatcaat atcaatcgta atggatgtca
2161 atttactttt agtgagcagt tttttaaaga ctttaaggca taatccacgc accacaacac
2221 accccacttg acaagtgata gccactatag tatattagtg gtgtggggta tgttgcaagt
2281 tccctaaagt gatagccaaa aggctacact ttagaacttg tgagggtggg caagcccccc
2341 ttcaatcccc ccttaaaaga ccgcaaatgt gcggtaattt gttgagtgat agccacatga
2401 aagagaggta ttttttatgt ttaatggaga acagcagaag aaagaaattg aaacaattcg
2461 agagagaaat atcacagtga aattgtcaga cgcagattgt gaaagactgg taagaaaatg
2521 cggagagcat gggttgacag tcggagagct gatagcgaat tttgtcggag atttggtagg
2581 tggtacttac tcaaacggaa gtgatgagag aatgtatgcc gaccagtggt ttgaccgctg
2641 ttggttcgga atgttcccag agcctacact tttgaatcac ttgctttgtt ggggctatga
2701 cccagaggac tatctggaca cgttggataa tatcgaaaca gcagtaaaag aaaaagagta
2761 ccttgcggaa cacccagaag aagcagacga agaagcacag tatcttgatg atgatattga
2821 gagctggcag gaagaactga aagacatgag agccgactgg aaaccagaga aagagccgaa
2881 catggacgaa gaacttgaac tcattaagaa gtgggttaag gaaagagagg acttaatcaa
2941 tgaataggac aagaccgaag cagatagtca taagggtgtc agaggaagaa cttgaagcca
3001 ttaagaaaaa agtggagcag tctggaaaga gccaacagca atatatcatt gaagccctta
3061 cacagaagca ggttgttaat ctggacggac tgaaagaaat atacccagaa ctgaaaaggc
3121 agggtaacaa cctcaaccag atagcgaaga aactcaatga aaatggatat gtagactata
3181 agcaggaact accaaacacc atgaaagaag tgagggaagt atggcagtta ttaaagcagt
3241 atcttcaaaa gcagggatag ggcaagccct tgactatgtg accaaagagg aaaagacaga
3301 ggagaagctt gtcagcggtc tgcattgtga agctgataca gtaaaggacg aaatgcaagc
3361 cactaaggag ctatggggca agactggggg cagaacctac aagcactttg ttcagagcta
3421 ccatgaggac gagcatatca caccagagca agcccataag aacgctatcg agcttgctaa
3481 gaatacagaa gcatggaaag ggcatgaggt gcttattgca acccatatag accgaggaca
3541 cattcactct cattttatcg tgaacagcgt gaattatgag gacggacaca agctacaatg
3601 gagcaatcaa gacctcaaag accttaaaga aaggtgcaac gagcagagca gagagcaagg
3661 gcttcacgta ccggaaaagg gcaagacgtt ctccggagaa gagagagagg aaacagttgc
3721 atggaacaag gacacatata acatcttgaa gcaggcagag caggggaaag taaagagcta
3781 tgtgcaggat atagcccttg cggttctgga ctgtaaggaa accgccacca gtcggcagga
3841 ctttatagaa cgcatggagc agaggggcta taagaccgac tggcaggaca accacaagta
3901 tattacctgg actgatttag ccagggaaaa cgcaggggaa aaggcttgta aaataaggga
3961 taacaagcta cagaagtatt acaatatgga ttttggaaag gaggaactgg agcgtgggtt
4021 tgaaatcaat gcacgaagca agcagacaga gcttgccaga gccgaagcag agcaacgagc
4081 aagagaacag cttgctagaa cagcaatccc agagaataac ggaactggaa caccaaatat
4141 cggagctttc ctcaatcaac tcaacgctga tgagcgagct tcagaagaaa agcgagataa

4201 tagtaaagct gaacgaaaag gaagagatat acaacaagag cgacttaatc aagaagcaga
4261 acgcagagct agagaagcag aacagagagc tagagaaagc aaggcaaaga gccgaagccg
4321 aagctatgac ggaggtagag accgttaaaa aagagtatgc agaaagggaa agagagcttg
4381 caaggacaca agccgaagcg aacaaggcaa agattgacgc agaggacacc agaagccacc
4441 agaaagagct tgtaaatgag aaagcccaac aaatgtataa cagcaggaga aaaagcctag
4501 aatcggctta tcagggcaag agaatagccc ttgacggagt tttctttggt tcgcttgcct
4561 atggggtact ttgtactgta ttcacagcag tgcgttcaga acgctttgta agcgatttta
4621 aggcattttt tggtactata tgggcttttc tattaacagc cttagaaaag ctcttacaac
4681 tggcaaaatg ggcttcacag ataggggata agataccgca ggagatagtt gcgtttatag
4741 tacactggct tgtgctgata gcggttgtcg ttctggtagg cggtggggca atcctgcttc
4801 tctttatcgg tgtagaatgg gtatatcaga attacaaaag tgattatgcc gacacaacaa
4861 gcctagctgt agcactggca agcctagcaa tatccgtatt ctttgcagag cctataaggg
4921 cggtaatccc tatcaatctt cttttattgc tgattattac ccacgtgtta tacgtgggtg
4981 ttaggtggtt cataaagggc tatagggaat ccagaggata ttggtaaagg tgcaaaaact
5041 cattgataag attgcggtca aacgaagcag gagaatatct tgtaaaaagc tcctgctttt
5101 tatataataa aaaaggtaaa aaaatctatt gataagatta gcggtcaagc gaagcaaggg
5161 gcagtatatt atataatata tctaagtagg tgaaaaaata tgcgtaggaa tgacaagaca
5221 agacatgaat ggagaaaggc atacaaggaa aaagaagagc tgatgattga aaggggatac
5281 ccagaagtaa gcccccatga cttctatcgt gagctgttcc cagagggtag cctgcagagc
5341 agggagcagg acggaaaagg caatatcata gcaacccaga taagaccatc tggaaagggc
5401 aggacaaaac agtgggtagt tgatgatagt ctggaaagac tggataaggt agtgggggac
5461 gagttcggac tcataccgcc tatatcgttc tatggcaaga gccacaccaa gaagaacgcc
5521 catgagttgt atgcggtggc aatagatatt gattatgtgg gaaagcaaca gctaaagaac
5581 ctcttaaagc agttcggaaa tggggtacag cttagaccga cctatcttgt gagcagtggc
5641 aagggggtac acctatatta cttcttgcag gaaccagtac agttatatca caatctggaa
5701 gataccttgt cagacctcaa agaagcattt ataaggcggt tgtggaacga tacaagcagt
5761 atcagaccag acagcccaga cataacggt atctatcaag gttttaggtg cgtaggcagt
5821 cagagcaagc taggggcaga atatccagta aaagcctata agctgtcaga aaaccgctat
5881 acactggaag atataaaggc aagcataccg aagtgtacag ttgatttgtc tggattgcag
5941 gagaagccga agcaggaaaa gagcaagctg tcactggaag aagccaagaa gctgtaccca
6001 gagtggtatc aagagagagt tgttgagggc agaccgaaga aaaaaggcac atgggtatgc
6061 aatcaagccc tttacgagtg gtggaaaggc aagataaacg gagaggtcaa agtaggcggt
6121 cggtacttct caatcatggc tttatgtgcc tatggcttga aatgtggcat accagaaagg
6181 cagataaggc aggacgcata ttcttttctg gaacatctgg aaagcctaac tgatgacgag
6241 gataaccact ttaccagaga ggacgtaaag gacgcattaa aagcccttaa agctgataac
6301 aagctacttt ctactatggc aagcagggaa tggatagaga agcagacaaa ggttgatata
6361 ccgccaaaca agcggaacgg aagaaagcaa gcaacacatt tgaagattgc aagaagtaca
6421 ctggctatta tgaatgagga cgagggaaaa gccttacaag gcagaccaga caaggcgaaa
6481 attgtcgaag aatggcaaaa aagccaccca gagggcagaa aagccgactg tattcgtgat
6541 actggattga gcaaaccgac agtgtatcgt tggtggaaag agggcgaagc cctttaagtc
6601 atttcccctt aattacccaa gagccataag aaaaagagtg tcaagggcag tcggaacgac
6661 tgtacattta cccttgatac tcttttttgt tggctacaat gccagagggg gaaacgactg
6721 tacgaccgtt ccgttacttc ctcatcggtt gcattgtcca ttccaaaaag agagagtaaa
6781 gggttgtctg cgacaatttt tttcactgct gccattcgct gatccgcaag cggaagctca
6841 tagctgcaga gaaaaaaaca agcccttgac tcaatgtcat taagttaagc aaaaacggcg
6901 cgcaagaaaa atgcccacct cgatttttcg aggtgggcat tttttatgct ttcgttatgt
6961 ggtttgcttg tgcttacttc gcgggcagga caatgacctg tccctcgtaa atggtgttgg
7021 cgttcacaag acggtcggcg ttgcgctcga agatggataa aataaaatag aggctataaa
7081 tagcctctat tttatgtgag aaatccctaa ataaaaagat gccagtgtgc tggaattcgc
7141 ccttaggctg cgctagggac ctctttagct ccttggaagc tgtcagtagt aggggatcgg
7201 tcttgccttg ctcgtcggtg atgtacttca ccagctccgc gaagtcgctc ttcttgatgg
7261 agcgcatggg gacgtgcttg gcaatcacgc gcaccccccg gccgttttag cggctaaaaa
7321 agtcatggct ctgccctcgg gcggaccacg cccatcatga ccttgccaag ctcgtcctgc
7381 ttctcttcga tcttcgccag cagggcgagg atcgtggcat caccgaaccg cgccgtgcgc
7441 gggtcgtcgg tgagccagag tttcagcagg ccgcccaggc ggcccaggtc gccattgatg
7501 cgggccagct cgcggacgtg ctcatagtcc acgacgcccg tgattttgta gccctggccg
7561 acggccagca ggtaggccga caggctcatg ccggccgccg ccgccttttc ctcaatcgct
7621 cttcgttcgt ctggaaggca gtacaccttg ataggtgggc tgcccttcct ggttggcttg
7681 gtttcatcag ccatccgctt gccctcatct gttacgccgg cggtagccgg ccagcctcgc
7741 agagcaggat tcccgttgag caccgccagg tgcgaataag ggacagtgaa gaaggaacac
7801 ccgctcgcgg gtgggcctac ttcacctatc ctgcccggct gacgccgttg gatacaccaa
7861 ggaaagtcta cacgaaccct ttggcaaaat cctgtatatc gtgcgaaaaa ggatggatat
7921 accgaaaaaa tcgctataat gaccccgaag caggaagggc gaattctgca gatctgaccg
7981 gtctctgaaa atataaaaac cacagattga tactaaaacc ttggttgtgt tgcttttcgg
8041 ggcttaaatc aaggaaaaat ccttgtttta agcctttcaa aaagaaacac aaggtctttg
8101 tactaacctg tggttatgta taaaattgta gattttaggg taacaaaaaa caccgtattt
8161 ctacgatgtt tttgcttaaa tacttgtttt tagttacaga caaacctgaa gttaactatt
8221 tatcaattcc tgcaattcgt ttacaaaacg gcaaatgtga aatccgtcac atactgcgtg

```
     8281 atgaacttga attgccaaag gaagtataat tttgttatct tctttataat atttccccat
     8341 agtaaaaata ggaatcaaat aatcatatcc tttctgcaaa ttcagattaa agccatcgaa
     8401 ggttgaccac ggtatcatag atacattaaa aatgttttcc ggagcatttg gctttccttc
     8461 cattctatga ttgtttccat accgttgcgt atcactttca taatctgcta aaaatgattt
     8521 aaagtcagac ttacactcag tccaaaggct ggaaaatgtt tcagtatcat tgtgaaatat
     8581 tgtatagctt ggtatcatct catcatatat ccccaattca ccatcttgat tgattgccgt
     8641 cctaaactct gaatggcggt ttacaatcat tgcaatataa taaagcattg caggatatag
     8701 tttcattccc ttttccttta tttgtgtgat atccacttta acggtcatgc tgtaggtaca
     8761 aggtacactt gcaaagtagt ggtcaaaata ctcttttctg ttccaactat ttttatcaat
     8821 tttttcaaat accatctaag ttccctctca aattcaagtt tatcgctcta atgaacaaag
     8881 atattatacc acatttttgt gaatttttca acttgcccac ttcgactgca ctcccgactt
     8941 aataacttct tgaacacttg ccgaaaaaga aaaactgccg ggtacgtacc cggggatcga
     9001 tccccggccg agcgctcttc cgcttcctcg ctcactgact cgctgcgctc ggtcgttcgg
     9061 ctgcggcgag cggtatcagc tcactcaaag gcggtaatac ggttatccac agaatcaggg
     9121 gataacgcag gaaagaacat gtgagcaaaa ggccagcaaa aggccaggaa ccgtaaaaag
     9181 gccgcgttgc tggcgttttt ccataggctc cgcccccctg acgagcatca caaaaatcga
     9241 cgctcaagtc agaggtggcg aaacccgaca ggactataaa gataccaggc gtttccccct
     9301 tgaagctccc tcgtgcgctc tcctgttccg accctgccgc ttaccggata cctgtccgcc
     9361 tttctccctt cgggaagcgt ggcgctttct catagctcac gctgtaggta tctcagttcg
     9421 gtgtaggtcg ttcgctccaa gctgggctgt gtgcacgaac cccccgttca gcccgaccgc
     9481 tgcgccttat ccggtaacta tcgtcttgag tccaacccgg taagacacga cttatcgcca
     9541 ctggcagcag ccactggtaa caggattagc agagcgaggt atgtaggcgg tgctacagag
     9601 ttcttgaagt ggtggcctaa ctacggctac actagaagga cagtatttgg tatctgcgct
     9661 ctgctgaagc cagttacctt cggaaaaaga gttggtagct cttgatccgg caaacaaacc
     9721 accgctggta gcggtggttt ttttgtttgc aagcagcaga ttacgcgcag aaaaaaagga
     9781 tctcaagaag atcctttgat cttttctacg gggtctgacg ctcagtggaa cgaaaactca
     9841 cgttaaggga ttttggtcat gaaatgcaag tttctaacta acatacatca tagttactaa
     9901 actatggtaa ctataatttt attaactata ttaagcactg attagtacta taactcaata
     9961 taagcatatc ccctgtatcg taactagaga accaaacgac ggaaaaagcg atatagataa
    10021 gtttagatag tgcaccaatt ttttactttt ctattttaga atctagcatt tccaatgctt
    10081 actaatacta tttttgattt ttaacttgtt ttaaaaaagc acctactaa
//
```

Supplementary File 2. GenBank formatted sequence of pIB80

```
LOCUS       pIB80                   8647 bp    DNA     circular SYN 19-DEC-2019
DEFINITION  Putative rep region of pCD-METRO cloned into pRPF185.
ACCESSION   pRPF-metro
KEYWORDS    .
SOURCE      Unknown
  ORGANISM  Unknown
            Unclassified.
REFERENCE   1  (bases 1 to 8647)
  AUTHORS   Boekhoud et al (2019)
  JOURNAL   Nature Communications
FEATURES             Location/Qualifiers
     rep_origin      complement(105..344)
                     /name="oriV"
     gene            complement(550..815)
                     /product="pseudogene; fragment of repA of pRPF185"
                     /standard_name="'repA"
     misc_feature    822..2874
                     /standard_name="Putative replicon pCD-METRO cloned
                     NcoI/NheI"
     CDS             complement(1305..2702)
                     /gene="IB136_3861"
                     /product="Putative replication protein"
                     /standard_name="ORF5"
     terminator      2880..2923
                     /standard_name="fdx terminator"
     CDS             complement(2932..3555)
                     /product="TetR repressor protein"
                     /standard_name="tetR"
     misc_feature    3577..3595
                     /note="Geneious type: operator"
                     /standard_name="tetO"
     misc_feature    3662..3680
                     /note="Geneious type: operator"
                     /standard_name="tetO"
     CDS             3747..5555
                     /product="beta-glucuronidase"
                     /standard_name="gusA"
     terminator      5615..5645
                     /standard_name="slpA terminator"
     misc_feature    complement(5943..6314)
                     /standard_name="traJ"
     CDS             complement(5943..6314)
                     /product="conjugation protein"
                     /standard_name="traJ"
     oriT            6347..6416
                     /name="origin of transfer"
     CDS             complement(6832..7455)
                     /gene="catP"
                     /product="chloramphenicol resistance protein"
                     /standard_name="catP"
ORIGIN
        1 gtgcaccaat tttttacttt tctattttag aatctagcat ttccaatgct tactaatact
       61 atttttgatt tttaacttgt tttaaaaaag cacctactaa ttaggattct agctctatat
      121 tctagttata cattctctat attctagctc tatattctag ttatacattc tctatattct
      181 agctctatat tctagttata cattctctat attctagctc tatattctag ttatacattc
      241 tctatattct agctctatat tctagttata cattctctat attctagctc tatattctag
      301 ttatacattc tctatattct agctctatat tctagttata cattctctaa aaaacatata
      361 tatatatact acatatatat gtacaattat tttaccaaaa gaaattttta aaacggctta
      421 atttcaatac tttgatgcca tttttttttt gcaattctcg tacaattatt ttaccaaaag
      481 aaatttttat taattagttt ttttgcataa aaataggact agaaaatcaa ttctaatcct
      541 atcaaaaatc tacttgttta atctatatgt atagtacata aatcctctat catttatttt
      601 agtttcccat acatctaaaa ttttagtatc taataaagat tctatcatat ttgaaattct
      661 ttgctgtgtt actcctaaat cttttgctaa ttcctcttga ttaattctta atatatttcc
      721 ttttatttta tcatctttga ccaataaatc ttgcttatat ctcatatgag tatataatct
      781 taattcttca gttgaaatta ttccccaaat tactgccatg gatcttgatt tgatgaagta
      841 caaggccatc ttcgagcgca acgccgaccg tcttgtgaac gccaacacca tttacgaggg
```

```
 901 acaggtcatt gtcctgcccg cgaagtaagc acaagcaaac cacataacga aagcataaaa
 961 aatgcccacc tcgaaaaatc gaggtgggca tttttcttgc gcgccgtttt tgcttaactt
1021 aatgacattg agtcaagggc ttgttttttt ctctgcagct atgagcttcc gcttgcggat
1081 cagcgaatgg cagcagtgaa aaaaattgtc gcagacaacc ctttactctc tctttttgga
1141 atggacaatg caaccgatga ggaagtaacg gaacggtcgt acagtcgttt ccccctctgg
1201 cattgtagcc aacaaaaaag agtatcaagg gtaaatgtac agtcgttccg actgcccttg
1261 acactctttt tcttatggct cttgggtaat taaggggaaa tgacttaaag ggcttcgccc
1321 tctttccacc aacgatacac tgtcggtttg ctcaatccag tatcacgaat acagtcggct
1381 tttctgccct ctgggtggct tttttgccat tcttcgacaa ttttcgcctt gtctggtctg
1441 ccttgtaagg cttttccctc gtcctcattc ataatagcca gtgtacttct tgcaatcttc
1501 aaatgtgttg cttgctttct tccgttccgc ttgtttggcg gtatatcaac ctttgtctgc
1561 ttctctatcc attccctgct tgccatagta gaaagtagct tgttatcagc tttaagggct
1621 tttaatgcgt cctttacgtc ctctctggta aagtggttat cctcgtcatc agttaggctt
1681 tccagatgtt ccagaaaaga atatgcgtcc tgccttatct gcctttctgg tatgccacat
1741 ttcaagccat aggcacataa agccatgatt gagaagtacc gaccgcctac tttgacctct
1801 ccgtttatct tgcctttcca ccactcgtaa agggcttgat tgcataccca tgtgcctttt
1861 ttcttcggtc tgccctcaac aactctctct tgataccact ctgggtacag cttcttggct
1921 tcttccagtg acagcttgct cttttcctgc ttcggcttct cctgcaatcc agacaaatca
1981 actgtacact tcggtatgct tgcctttata tcttccagtg tatagcggtt ttctgacagc
2041 ttataggctt ttactggata ttctgcccct agcttgctct gactgcctac gcacctaaaa
2101 ccttgataga tacccgttat gtctgggctg tctggtctga tactgcttgt atcgttccac
2161 aaccgcctta taaatgcttc tttgaggtct gacaaggtat cttccagatt gtgatataac
2221 tgtactggtt cctgcaagaa gtaatatagg tgtacccccct tgccactgct cacaagatag
2281 gtcggtctaa gctgtacccc atttccgaac tgctttaaga ggttctttag ctgttgcttt
2341 cccacataat caatatctat tgccaccgca tacaactcat gggcgttctt cttggtgtgg
2401 ctcttgccat agaacgatat aggcggtatg agtccgaact cgtcccccac taccttatcc
2461 agtctttcca gactatcatc aactacccac tgttttgtcc tgccctttcc agatggtctt
2521 atctgggttg ctatgatatt gccttttccg tcctgctccc tgctctgcag gctaccctct
2581 gggaacagct cacgatagaa gtcatggggg cttacttctg ggtatcccct ttcaatcatc
2641 agctcttctt tttccttgta tgcctttctc cattcatgtc ttgtcttgtc attcctacgc
2701 atattttttc acctacttag atatattata taatatactg ccccttgctt cgcttgaccg
2761 ctaatcttat caatagattt ttttaccttt tttattatat aaaaagcagg agctttttac
2821 aagatattct cctgcttcgt ttgaccgcaa tcttatcaat gagtttttgc acctgctagc
2881 ataaaaataa gaagcctgca tttgcaggct tcttattttt atggtacctt aagacccact
2941 ttcacattta agttgttttt ctaatccgca tatgatcaat tcaaggccga ataagaaggc
3001 tggctctgca ccttggtgat caaataattc gatagcttgt cgtaataatg gcggcatact
3061 atcagtagta ggtgtttccc tttcttcttt agcgacttga tgctcttgat cttccaatac
3121 gcaacctaaa gtaaaatgcc ccacagcgct gagtgcatat aatgcattct ctagtgaaaa
3181 accttgttgg cataaaaagg ctaattgatt ttcgagagtt tcatactgtt tttctgtagg
3241 ccgtgtacct aaatgtactt ttgctccatc gcgatgactt agtaaagcac atctaaaact
3301 tttagcgtta ttacgtaaaa aatcttgcca gctttcccct tctaaagggc aaaagtgagt
3361 atggtgccta tctaacatct caatggctaa ggcgtcgagc aaagcccgct tatttttttac
3421 atgccaatac aatgtaggct gctctacacc tagcttctgg gcgagtttac gggttgttaa
3481 accttcgatt ccgacctcat taagcagctc taatgcgctg ttaatcactt tacttttatc
3541 taatctagac atcattaatt cctccttttt gttgacatta tatcattgat agagttattt
3601 gtcaaactag ttttttattt cgatgccctg gacttcatga aaaactaaaa aaaatattga
3661 cactctatca ttgatagagt ataattaaaa taagcttgat cgtagcgtta acagatctga
3721 gctcctgcag taaaggagaa aattttatgt tacgtcctgt agaaacccca acccgtgaaa
3781 tcaaaaaact cgacggcctg tgggcattca gtctggatcg cgaaaactgt ggaattgatc
3841 agcgttggtg ggaaagcgcg ttacaagaaa gccgggcaat tgctgtgcca ggcagtttta
3901 acgatcagtt cgccgatgca gatattcgta attatgcggg caacgtctgg tatcagcgcg
3961 aagtctttat accgaaaggt tgggcaggcc agcgtatcgt gctgcgtttc gatgcggtca
4021 ctcattacgg caaagtgtgg gtcaataatc aggaagtgat ggagcatcag ggcggctata
4081 cgccatttga agccgatgtc acgccgtatg ttattgccgg gaaaagtgta cgtatcaccg
4141 tttgtgtgaa caacgaactg aactggcaga ctatcccgcc gggaatggtg attaccgacg
4201 aaaacggcaa gaaaaagcag tcttacttcc atgatttctt taactatgcc gggatacatc
4261 gcagcgtaat gctctacacc acgccgaaca cctgggtgga cgatatcacc gtggtgacgc
4321 atgtcgcgca agactgtaac cacgcgtctg ttgactggca ggtggtggcc aatggtgatg
4381 tcagcgttga actgcgtgat gcggatcaac aggtggttgc aactggacaa ggcactagcg
4441 ggactttgca agtggtgaat ccgcacctct ggcaaccggg tgaaggttat ctctatgaac
4501 tgtgcgtcac agccaaaagc cagacagagt gtgatatcta cccgcttcgc gtcggcatcc
4561 ggtcagtggc agtgaagggc gaacagttcc tgattaacca caaaccgttc tactttactg
4621 gctttggtcg tcatgaagat gcggacttgc gtggcaaagg attcgataac gtgctgatgg
4681 tgcacgacca cgcattaatg gactggattg gggccaactc ctaccgtacc tcgcattacc
4741 cttacgctga agagatgctc gactgggcag atgaacatgg catcgtggtg attgatgaaa
4801 ctgctgctgt cggctttaac ctctctttag gcattggttt cgaagcgggc aacaagccga
4861 aagaactgta cagcgaagag gcagtcaacg gggaaactca gcaagcgcac ttacaggcga
4921 ttaaagagct gatagcgcgt gacaaaaacc acccaagcgt ggtgatgtgg agtattgcca
```

```
4981 acgaaccgga tacccgtccg caaggtgcac gggaatattt cgcgccactg gcggaagcaa
5041 cgcgtaaact cgacccgacg cgtccgatca cctgcgtcaa tgtaatgttc tgcgacgctc
5101 acaccgatac catcagcgat ctctttgatg tgctgtgcct gaaccgttat tacggatggt
5161 atgtccaaag cggcgatttg gaaacggcag agaaggtact ggaaaaagaa cttctggcct
5221 ggcaggagaa actgcatcag ccgattatca tcaccgaata cggcgtggat acgttagccg
5281 ggctgcactc aatgtacacc gacatgtgga gtgaagagta tcagtgtgca tggctggata
5341 tgtatcaccg cgtctttgat cgcgtcagcg ccgtcgtcgg tgaacaggta tggaatttcg
5401 ccgattttgc gacctcgcaa ggcatattgc gcgttggcgg taacaagaaa gggatcttca
5461 ctcgcgaccg caaaccgaag tcggcggctt ttctgctgca aaaacgctgg actggcatga
5521 acttcggtga aaaaccgcag cagggaggca aacaatgaat caacaactct cctggcgcac
5581 cggatcctat aagttttaat aaaactttaa atagaaaaag gcttctctca tgagaagtct
5641 tttttattta aaataaatat aaaataaaat agaggctata aatagcctct attttatgtg
5701 agaaatccct aaataaaaag atgccagtgt gctggaattc cccttcaata ttccacaata
5761 aggctgtagg cgctagggac ctctttagct ccttggaagc tgtcagtagt aggggatcgg
5821 tcttgccttg ctcgtcggtg atgtacttca ccagctccgc gaagtcgctc ttcttgatgg
5881 agcgcatggg gacgtgcttg gcaatcacgc gcaccccccg gccgttttag cggctaaaaa
5941 agtcatggct ctgccctcgg gcggaccacg cccatcatga ccttgccaag ctcgtcctgc
6001 ttctcttcga tcttcgccag cagggcgagg atcgtggcat caccgaaccg cgccgtgcgc
6061 gggtcgtcgg tgagccagag tttcagcagg ccgcccaggc ggcccaggtc gccattgatg
6121 cgggccagct cgcggacgtg ctcatagtcc acgacgcccg tgattttgta gccctggccg
6181 acggccagca ggtaggccga caggctcatg ccggccgccg ccgccttttc ctcaatcgct
6241 cttcgttcgt ctggaaggca gtacaccttg ataggtgggc tgcccttcct ggttggcttg
6301 gtttcatcag ccatccgctt gccctcatct gttacgccgg cggtagccgg ccagcctcgc
6361 agagcaggat tcccgttgag caccgccagg tgcgaataag ggacagtgaa gaaggaacac
6421 ccgctcgcgg gtgggcctac ttcacctatc ctgcccggct gacgccgttg gatacaccaa
6481 ggaaagtcta cacgaaccct ttggcaaaat cctgtatatc gtgcgaaaaa ggatggatat
6541 accgaaaaaa tcgctataat gaccccgaag caggaagggc gaattctgca gatctgaccg
6601 gtctctgaaa atataaaaac cacagattga tactaaaacc ttggttgtgt tgcttttcgg
6661 ggcttaaatc aaggaaaaat ccttgtttta agcctttcaa aaagaaacac aaggtctttg
6721 tactaacctg tggttatgta taaaattgta gattttaggg taacaaaaaa caccgtattt
6781 ctacgatgtt tttgcttaaa tacttgtttt tagttacaga caaacctgaa gttaactatt
6841 tatcaattcc tgcaattcgt ttacaaaacg gcaaatgtga aatccgtcac atactgcgtg
6901 atgaacttga attgccaaag gaagtataat tttgttatct tctttataat atttccccat
6961 agtaaaaata ggaatcaaat aatcatatcc tttctgcaaa ttcagattaa agccatcgaa
7021 ggttgaccac ggtatcatag atacattaaa aatgttttcc ggagcatttg gctttccttc
7081 cattctatga ttgtttccat accgttgcgt atcactttca taatctgcta aaaatgattt
7141 aaagtcagac ttacactcag tccaaaggct ggaaaatgtt tcagtatcat tgtgaaatat
7201 tgtatagctt ggtatcatct catcatatat ccccaattca ccatcttgat tgattgccgt
7261 cctaaactct gaatggcggt ttacaatcat tgcaatataa taaagcattg caggatatag
7321 tttcattccc ttttccttta tttgtgtgat atccacttta acggtcatgc tgtaggtaca
7381 aggtacactt gcaaagtagt ggtcaaaata ctcttttctg ttccaactat ttttatcaat
7441 tttttcaaat accatctaag ttccctctca aattcaagtt tatcgctcta atgaacaaag
7501 atattatacc acatttttgt gaatttttca acttgcccac ttcgactgca ctcccgactt
7561 aataacttct tgaacacttg ccgaaaaaga aaaactgccg ggtacgtacc cgggatcgat
7621 ccccgccgag cgctcttccg cttcctcgct cactgactcg ctgcgctcgg tcgttcggct
7681 gcggcgagcg gtatcagctc actcaaaggc ggtaatacgg ttatccactg aatcagggga
7741 taacgcagga aagaacatgt gagcaaaagg ccagcaaaag gccaggaacc gtaaaaaggc
7801 cgcgttgctg gcgtttttcc ataggctccg cccccctgac gagcatcaca aaaatcgacg
7861 ctcaagtcag aggtggcgaa acccgacagg actataaaga taccaggcgt ttccccctgg
7921 aagctccctc gtgcgctctc ctgttccgac cctgccgctt accggatacc tgtccgcctt
7981 tctcccttcg ggaagcgtgg cgctttctca atgctcacgc tgtaggtatc acagttcggt
8041 gtaggtcgtt cgctccaagc tgggctgtgt gcacgaaccc cccgttcagc ccgaccgctg
8101 cgccttatcc ggtaactatc gtcttgagtc caacccggta agacacgact tatcgccact
8161 ggcagcagcc actggtaaca ggattagcag agcgaggtat gtaggcggtg ctacagagtt
8221 cttgaagtgg tggcctaact acggctacac tagaaggaca gtatttggta tctgcgctct
8281 gctgaagcca gttaccttcg gaaaaagagt tggtagctct tgatccggca aacaaaccac
8341 cgctggtagc ggtggttttt ttgtttgcaa gcagcagatt acgcgcagaa aaaaaggatc
8401 tcaagaagat cctttgatct tttctacggg gtctgacgct cagtggaacg aaaactcacg
8461 ttaagggatt ttggtcatga aatgcaagtt tctaactaac atacatcata gttactaaac
8521 tatggtaact ataattttat taactatatt aagcactgat tagtactata actcaatata
8581 agcatatccc ctgtatcgta actagagaac caaacgacgg aaaaagcgat atagataagt
8641 ttagata
//
```

References

1. O'Connor JR, Galang MA, Sambol SP, et al. Rifampin and rifaximin resistance in clinical isolates of Clostridium difficile. *Antimicrobial agents and chemotherapy* 2008; **52**(8): 2813-7.
2. Brouwer MS, Allan E, Mullany P, Roberts AP. Draft genome sequence of the nontoxigenic Clostridium difficile strain CD37. *J Bacteriol* 2012; **194**(8): 2125-6.
3. Purdy D, O'Keeffe TA, Elmore M, et al. Conjugative transfer of clostridial shuttle vectors from Escherichia coli to Clostridium difficile through circumvention of the restriction barrier. *Molecular microbiology* 2002; **46**(2): 439-52.
4. Khodadoost L, Hussain H, Mullany P. Plasmids can transfer to Clostridium difficile CD37 and 630Deltaerm both by a DNase resistant conjugation-like mechanism and a DNase sensitive mechanism. *FEMS microbiology letters* 2017; **364**(21).
5. EUCAST. European Committee on Antimicrobial Susceptibility Testing. Breakpoint tables for interpretation of MICs and zone diameters, version 8.0, 2018. 2018. http://www.eucast.org/clinical_breakpoints/.
