# Supplementary material for: Plasmid-mediated metronidazole resistance in Clostridioides difficile
Source: Nat Commun. 2020 Jan 30;11:598. doi: 10.1038/s41467-020-14382-1 (PMC6992631; doi:10.1038/s41467-020-14382-1)
Supplement: Supplementary file 5 — Reporting Summary [file 41467_2020_14382_MOESM5_ESM.pdf]

nature research

Corresponding author(s): W.K. Smits, PhD

Last updated by author(s): Dec 19, 2019

Reporting Summary

Nature Research wishes to improve the reproducibility of the work that we publish. This form provides structure for consistency and transparency in reporting. For further information on Nature Research policies, see Authors & Referees and the Editorial Policy Checklist.

Statistics

For all statistical analyses, confirm that the following items are present in the figure legend, table legend, main text, or Methods section.

n/a	Confirmed	
☐	☒	The exact sample size (*n*) for each experimental group/condition, given as a discrete number and unit of measurement
☐	☒	A statement on whether measurements were taken from distinct samples or whether the same sample was measured repeatedly
☐	☒	The statistical test(s) used AND whether they are one- or two-sided<br>*Only common tests should be described solely by name; describe more complex techniques in the Methods section.*
☒	☐	A description of all covariates tested
☒	☐	A description of any assumptions or corrections, such as tests of normality and adjustment for multiple comparisons
☐	☒	A full description of the statistical parameters including central tendency (e.g. means) or other basic estimates (e.g. regression coefficient) AND variation (e.g. standard deviation) or associated estimates of uncertainty (e.g. confidence intervals)
☐	☒	For null hypothesis testing, the test statistic (e.g. *F*, *t*, *r*) with confidence intervals, effect sizes, degrees of freedom and *P* value noted<br>*Give P values as exact values whenever suitable.*
☒	☐	For Bayesian analysis, information on the choice of priors and Markov chain Monte Carlo settings
☒	☐	For hierarchical and complex designs, identification of the appropriate level for tests and full reporting of outcomes
☒	☐	Estimates of effect sizes (e.g. Cohen's *d*, Pearson's *r*), indicating how they were calculated

Our web collection on statistics for biologists contains articles on many of the points above.

Software and code

Policy information about availability of computer code

Data collection	No software was used.
Data analysis	Cutadapt v1.2.1, prinseq-lite v0.20.0, Spades v3.10.1, IDBA_UD v1.1.1, Ray v2.3.0, Velvet v1.2.10, Edena v3.131028, kmergenie v1.6741, Bowtie2 v2.3.1, Samtools v1.5, Blastn v2.6, Picard tools package v1.94, GapFiller v1.11, kmerspectrumanalyzer August 2013, Jellyfish v1.1.11. 16, bedtools genomecov v2.2.16, prodigal v2.6.3, Rnammer v1.2, Aragorn v1.2.38, CRISPR recognition tool v1.2, InterproScan v5.26-65.0, PRIAM March 2015, legacy blast v2.2.26, dbCAN v5.0, BCFtools v1.1-134, SRA toolkit v2.8.2-1, BioRad GelDoc XR, Adobe Photoshop CC 2018, Adobe Illustrator CC 2018, Artemis and DNAplotter Release 17.0.1.

For manuscripts utilizing custom algorithms or software that are central to the research but not yet described in published literature, software must be made available to editors/reviewers. We strongly encourage code deposition in a community repository (e.g. GitHub). See the Nature Research guidelines for submitting code & software for further information.

Data

Policy information about availability of data

All manuscripts must include a data availability statement. This statement should provide the following information, where applicable:

- Accession codes, unique identifiers, or web links for publicly available datasets
- A list of figures that have associated raw data
- A description of any restrictions on data availability

All sequence data generated in this study has been uploaded to the European Nucleotide Archive under project PRJEB24167 with accession numbers ERR2232520-ERR2232537 and ERR3611150-ERR3611153. The genome assembly for IB136, including the annotated sequence of pCD-METRO, can be found under accession number ERZ807316.The source data underlying Figs 1, 2b, 2c, 3, 4, 5, 6, 7 and 8 and Supplemental Figures 2 are provided as a Source Data file.

Field-specific reporting

Please select the one below that is the best fit for your research. If you are not sure, read the appropriate sections before making your selection.

☒ Life sciences ☐ Behavioural & social sciences ☐ Ecological, evolutionary & environmental sciences

For a reference copy of the document with all sections, see nature.com/documents/nr-reporting-summary-flat.pdf

Life sciences study design

All studies must disclose on these points even when the disclosure is negative.

Sample size	No sample size calculation was performed. Triplicate values were used to allow for ANOVA and Tukey's Test.
Data exclusions	N/A
Replication	All attempts at replication were succesful.
Randomization	N/A
Blinding	N/A

Reporting for specific materials, systems and methods

We require information from authors about some types of materials, experimental systems and methods used in many studies. Here, indicate whether each material, system or method listed is relevant to your study. If you are not sure if a list item applies to your research, read the appropriate section before selecting a response.

Materials & experimental systems

n/a	Involved in the study	
☒	☐	Antibodies
☒	☐	Eukaryotic cell lines
☒	☐	Palaeontology
☒	☐	Animals and other organisms
☐	☒	Human research participants
☒	☐	Clinical data

Methods

n/a	Involved in the study	
☒	☐	ChIP-seq
☒	☐	Flow cytometry
☒	☐	MRI-based neuroimaging

Human research participants

Policy information about studies involving human research participants

Population characteristics	The Netherlands Donor Feces Bank (NDFB) is a national approved facility in the Leden University Medical Centre for treatment of patients with muliple recurrent Clostridioides difficile infections (rCDI) with feces microbiota transplantations. The NDFB is headed by prof. dr. Ed Kuijper and dr. Josbert Keller and falls under responsibillity of the LUMC.
Recruitment	Requests for treatment of patients with rCDI are submitted to the NDFB and are evaluated by a team of experts. Patients are also asked to sign an informed consent in which they agree with scientific research of stool samples to study the effect of FMT on the microbiota composition and eradication of C. difficile.
Ethics oversight	All studies and informed consent documents have been approved by the Medical Ethical Commitee of the Leiden Medical University Centre.

Note that full information on the approval of the study protocol must also be provided in the manuscript.
