# Supplementary material for: Plasmid-mediated metronidazole resistance in Clostridioides difficile
Source: Nat Commun. 2020 Jan 30;11:598. doi: 10.1038/s41467-020-14382-1 (PMC6992631; doi:10.1038/s41467-020-14382-1)
Supplement: Supplementary file 6 — Description of Additional Supplementary Files [file 41467_2020_14382_MOESM6_ESM.pdf]

Description of Additional Supplementary Files

File name: Supplementary Data 1
Description: Strains characterized with agar dilution and tested for presence of pCD-METRO

File name: Supplementary Data 2
Description: Assembly of the MTZ^R RT020 strain IB136
